# The noncoding RNA HOXD-AS1 is a critical regulator of the metastasis and apoptosis phenotype in human hepatocellular carcinoma

**DOI:** 10.1186/s12943-017-0676-x

**Published:** 2017-07-19

**Authors:** Shan Lu, Jiansheng Zhou, Yimin Sun, Nan Li, Mingyong Miao, Binghua Jiao, Huan Chen

**Affiliations:** 1 Department of Biochemistry and Molecular Biology, Second Military Medical University, 800 Xiangyin Road, Shanghai, 200433 China; 2 State Key Laboratory of Proteomics, Beijing Proteome Research Center, Beijing Institute of Radiation Medicine, Beijing, 102206 China; 3 National Engineering Research Center for Beijing Biochip Technology, 18 Life Science Parkway, Beijing, 102206 China; 4 The Third Department of Hepatic Surgery, Eastern Hepatobiliary Hospital, Second Military Medical University, Shanghai, 200433 China

**Keywords:** Human hepatocellular carcinoma, HOXD-AS1, ARHGAP11A, miR19a, lncRNA

## Abstract

**Background:**

Despite accumulating evidence that long noncoding RNAs (lncRNAs) are associated with cancer development in multiple types of cancer, the biological roles of many lncRNAs in human hepatocellular carcinoma (HCC) metastasis have not been well characterized.

**Methods:**

A lncRNA+ mRNA human gene expression microarray analysis was used to identify differentially expressed lncRNAs in metastatic HCC tissues compared to non-metastatic tissue.

**Results:**

We observed remarkable overexpression of HOXD-AS1 in metastatic cancer tissues. In vitro and in vivo gain- or loss-of-function studies re-affirmed that HOXD-AS1 is able to facilitate cancer metastasis and inhibit apoptosis. Moreover, we identified that HOXD-AS1 upregulated the Rho GTPase activating protein 11A (ARHGAP11A) by competitively binding to microRNA-19a (miR19a), resulting in induced metastasis. Interestingly, the regulator of G-protein signaling 3 (RGS3), a potential inhibitor of the MEK-ERK1/2 signaling axis, was also found to be downregulated by ectopic HOXD-AS1 overexpression, leading to a remarkably reduced apoptotic effect.

**Conclusions:**

The present investigation strongly indicates that HOXD-AS1 is an oncogenic lncRNA that promotes HCC metastasis and that its pro-metastatic phenotype can partially be attributed to the HOXD-AS1/miR19a/ARHGAP11A signaling axis.

**Electronic supplementary material:**

The online version of this article (doi:10.1186/s12943-017-0676-x) contains supplementary material, which is available to authorized users.

## Background

Hepatocellular carcinoma (HCC) is the sixth most prevalent cancer and one of the leading causes of cancer-related death in both men and woman. In 2008, HCC resulted in the deaths of approximately 700,000 people worldwide, with approximately half of these deaths occurring in China [1, 2]. Despite current knowledge and scientific advances in diagnosis and treatment modalities, the long-term survival rate of HCC still remains dismal. The unfavorable outcome could be attributed to two problems causing recurrence, intrahepatic metastasis and/or the development of de novo tumors in the remnant liver. However, the underlying biological mechanisms may differ greatly [3–5]. In particular, the molecular risk factors contributing to intrahepatic metastasis and early recurrence after hepatectomy have not been well characterized.

It is well-known that cancer metastasis is a complex multistep process that involves a myriad of genetic alterations. Previous studies have identified that signaling pathways such as transforming growth factor-β (TGF-β), vascular endothelial growth factor (VEGF), Wnt/β-catenin, MAPK and small G-protein signaling play important roles in mediating HCC development [6–10]. Recently, more functional players have been uncovered and are now being integrated to explain HCC initiation and progression, including microRNAs (miRNAs), long non-coding RNAs (lncRNAs) and epigenetic factors [11–14].

LncRNAs are a group of poorly conserved endogenous RNA molecules longer than 200 nt in length. In the past few years, several major reports have highlighted the importance of lncRNAs in HCC metastasis, suggesting the involvement of lncRNAs in cell signaling and cancer progression. For instance, metastasis-associated lung adenocarcinoma transcript-1 (MALAT1) and lncRNA-activated by TGF-β (lncRNA-ATB) are upregulated in HCC tissues, while lncRNA low expression in tumor (lncRNA-LET) and lncRNA downregulated expression by HBx (lncRNA-Dreh) are downregulated in HCC. Furthermore, in vitro or in vivo studies have confirmed that the aberrant expression of these lncRNAs is associated with hepatoma cell proliferation or invasion [15–19]. Interestingly, two lncRNAs within the Class I homeobox genes (HOX) chromosomal loci, namely, the hox transcript antisense intergenic RNA (HOTAIR) and the HOXA transcript at the distal tip (HOTTIP), were identified as being correlated with the risk of recurrence and the predicted outcomes in HCC patients [14, 20].

In mammals, 39 HOX genes cluster on four chromosomal loci, named HOXA through HOXD, and are important for body patterning coordination during embryonic development [21, 22]. Currently, 231 HOX-related ncRNAs have been identified including HOTAIR and HOTTIP. In this study, we report that the lncRNA HOXD cluster antisense RNA 1 (HOXD-AS1), which is expressed on the HOXD locus located on chromosome 2q31.2, plays a crucial role in HCC progression and is associated with metastasis and apoptosis phenotypes in cancer cells.

## Methods

### Clinical materials

Fifty cancerous and adjacent noncancerous specimens were obtained from patients with informed consent who underwent surgery for primary HCC at the Eastern Hepatobiliary Surgery Hospital (Shanghai, China) between 2010 and 2013. The study was approved by the Committees for the Ethical Review of Research involving Human Subjects from the Second Military Medical University. Among the patients, 27 had primary HCC lesions accompanied by intrahepatic metastasis at surgery (with tumor emboli in the major branches of the portal vein) and 23 had solitary HCC with no metastasis or recurrence during the two-year follow-up; the two groups were defined as the metastatic and non-metastatic groups. LncRNA and mRNA gene expression profiles were generated from six primary HCCs (three from the metastatic group and three from the non-metastatic group) and from the corresponding noncancerous hepatic fresh frozen tissues.

### LncRNA microarray analysis

The LncRNA Human Gene Expression Microarray V4.0 (CapitalBio Corp, Beijing, China) was used. In brief, double-stranded cDNAs were synthesized, purified and eluted. Complementary RNA was synthesized from the eluted dsDNA products using a T7 Enzyme Mix. After amplification, the cDNA products were purified and labelled. Array hybridization was performed in a CapitalBio BioMixerTM II Hybridization Station overnight and washed. Slides were scanned and the microarray image information was converted into spot intensity values. The signal after background subtraction was exported directly into GeneSpring software for quartile normalization and further data analysis. We selected differentially expressed lncRNAs according to the following criteria: fold change >2 and *P* < 0.05. Hierarchical clustering analysis was employed on differentially expressed lncRNAs.

### Cell culture and transfection

HCCLM3, MHCC97H, MHCC97L, SMMC7721 and L02 cells were cultured in DMEM (Biowest, Loire, France) with 10% fetal bovine serum (FBS, Biowest, Loire, France) in a humidified atmosphere containing 5% CO_2_ at 37 °C. HOXD-AS1 overexpression and control pcDNA3.1 plasmids and siRNAs (GenePharma, Shanghai, China, Additional file 1: Table S1) were transfected using Lipofectamine 2000 (Invitrogen, CA, USA) according to the manufacturer’s protocols.

### RNA extraction, reverse transcription and QRT-PCR

Total RNA was isolated from the prepared liver samples and cells using TRIzol reagent (Invitrogen, Carlsbad, CA, USA). Complementary DNA was synthesized following the manufacturer’s protocol (MBI Fermentas, Vilnius, Lithuania). QRT-PCR was performed with a standard SYBR-green PCR kit (TOYOBO, Osaka, Japan), and gene-specific PCR amplification was performed using the ABI 7300 (Applied Biosystems, Darmstadt, Germany). The primers are listed in Additional file 1: Table S2.

### Analysis of cell motility

Cell motility was monitored using transwell and wound scratch assays. Briefly, 1 × 10^4^ plasmid or siRNA-treated HCCLM3cells were added to the upper chamber and allowed to migrate through the polycarbonate membrane (8.0 μm PET, Millipore, Bedford, MA). After 24 h, the cells that had migrated to the lower chamber were fixed and stained with crystal violet. The wound scratch assay was performed using a pipette tip to scratch the cell layer 24 h after transfection, and phase contrast images of the wounds were recorded after 0 and 48 h.

### MTT assay for cell proliferation

After transfection, cells were plated in 96-well plates at a density of 4 × 10^3^ cells/well and incubated for the indicated times. At the end of incubation, 10 μl MTT (5 mg/mL, Sigma, USA) was added to each well, and the cells were incubated for 4 h. After staining, the samples were dissolved in DMSO, and the absorbance was recorded at 595 nm.

### Colony formation assay

After treatment, cells were re-seeded in 6-well plates at a density of 500 cells/well and cultured to form nature colonies. After 10 days, the cells were washed with PBS twice and fixed with 4% paraformaldehyde for 20 min. The fixed colonies were stained by crystal purple for 10 min, photographed and counted.

### Construction of stable cell lines with overexpressed HOXD-AS1 and the mouse xenograft model

To observe the effects of HOXD-AS1 overexpression on growth and metastasis in vivo, luciferase tagged cancer cells were stably infected with lentiviruses encoding HOXD-AS1 with puromycin selection. Tumor cell inoculation into the nude mice were performed as described in previously [19]. To investigate experimental lung metastasis or liver metastasis, the anesthetized nude mouse were inoculated with different stable cell lines by tail vein injection or intra-spleen injection. Four weeks after tail vein injection or six weeks after intra-spleen injection of HCCLM3 cells, lung metastases and liver metastases were monitored by using the IVIS@ Lumina II system. Error bars show standard deviation. For tumor growth evaluation, the skin along back of mouse is incised and injected with 1 × 10^7^ tumor cells. After 4 weeks the tumors in mice were removed, photographed and determined by tumor weight and tumor volumn. The animal studies were approved by the Institutional Animal Care and Use Committee of the Second Military Medical University, Shanghai, China.

### Apoptosis analysis

Apoptosis was analyzed by flow cytometry using the Annexin V-PI detection kit. After transfection, cells were treated with doxorubicin (Dox, 1 μM) for 24 h and then harvested for Annexin V-PI staining according to the manufacturer’s instructions (BD Biosciences PharMingen). The double-stained cells were analyzed by flow cytometry, and the early or late apoptotic cells were measured.

### Luciferase reporter assay

The 3′-UTR region of the Rho GTPase activating protein 11A (ARHGAP11A), which contains the miR-19a response element, was cloned into the pGL4.13 luciferase reporter vector to generate the luc vector. The miR-19a binding site in the luc vector was mutated to generate the luc mutant vector. To confirm the regulatory relationship between miR-19a and ARHGAP11A, miR-19a mimics, mimic NC, pcDNA3.1-HOXD-AS1, pcDNA3.1-HOXD-AS1-mut (miR-19a binding site mutation) or empty vectors were transfected into HCCLM3 cells. Forty-eight hours later, all protein extracts were analyzed using the dual luciferase reporter assay system (Promega).

### Westernblot analysis

Cell samples were lysed in RIPA lysis buffer and centrifuged at 12,000 rpm at 4 °C for 15 min. Equal amounts of protein were separated on a gel and transferred to PVDF membranes (Millipore). The membranes were incubated with antibodies specific for caspase 3, caspase 9, PARP, phospho-ERK, phospho-MEK (Cell Signaling Technology, Danvers, MA, USA) and GAPDH (Epitomics, Burlingame, CA). The immunoblotting sample was incubated with horseradish peroxidase (HRP)-coupled anti-rabbit secondary antibodies (Santa Cruz, CA, USA) and visualized using enhanced chemiluminescence (Pierce, Rockford, USA).

### Statistical analysis

For statistical analysis, Student’s t test was used for parametric variables; chi-square test and Fisher’s exact test (two-tailed) were used for nonparametric variables. Disease-free survival (DFS) in patients from The Cancer Genome Atlas (TCGA) dataset was analyzed using the Kaplan-Meier method and using the Gehan-Breslow-Wilcoxon test or the log-rank test for univariate analysis. All tests were performed at least three times, and a *P* value of less than 0.05 was considered statistically significant.

## Results

### HOXD-AS1 is upregulated in Metastatic HCC tissue and is associated with metastatic phenotypes in HCC cells

**Fig. 1:**
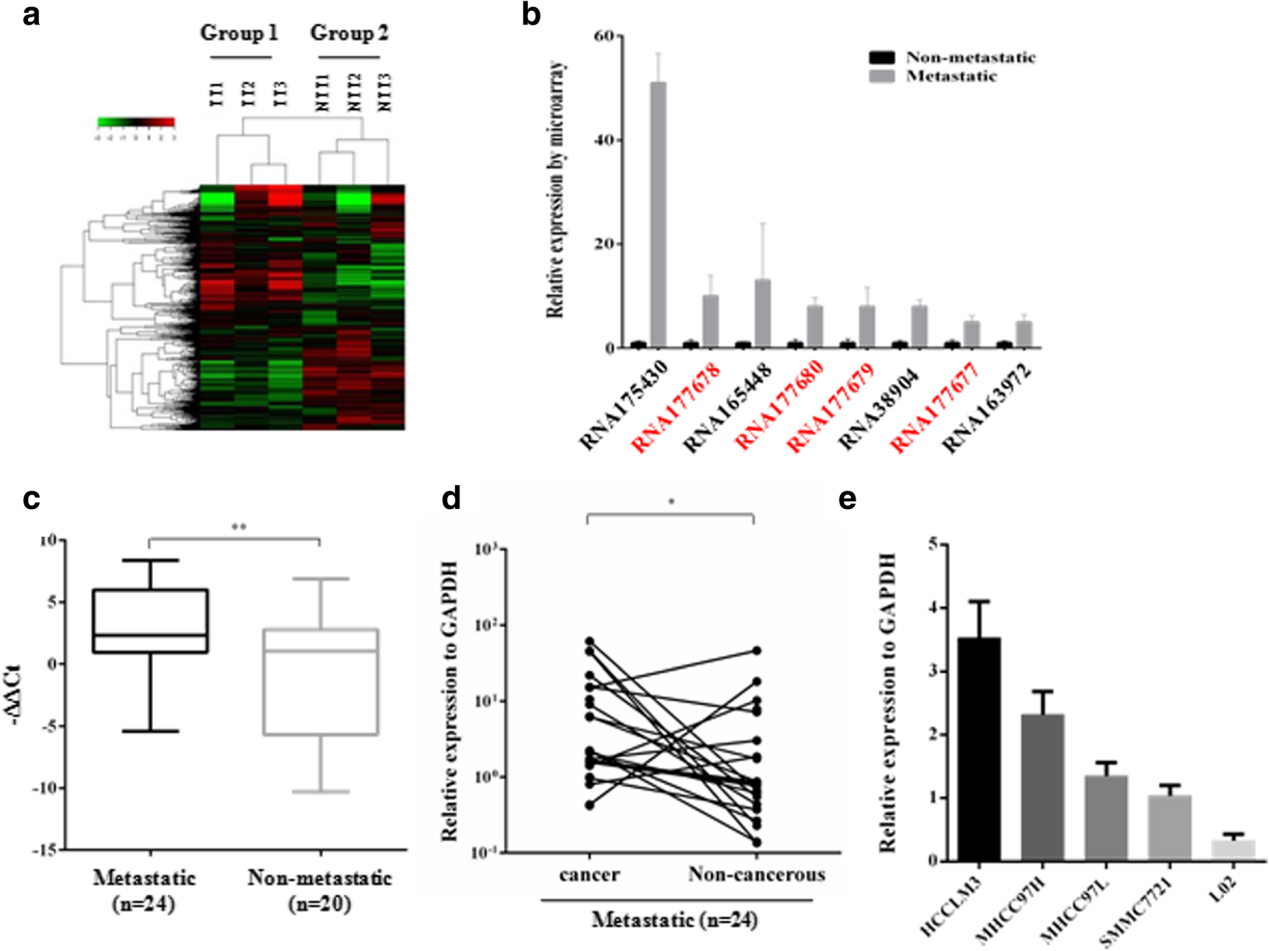
HOXD-AS1 is involved in HCC metastasis. **a** LncRNA + mRNA expression profiles were generated from two groups of HCC tissue samples. Three samples in the metastatic group (Group 1) were from patients who had intrahepatic metastasis at the time of surgery. For the non-metastatic group (Group 2), three samples were from HCC patients with no metastasis or recurrence during the two-year follow-up. **b** Top-ranked upregulated lncRNAs detected by the lncRNA + mRNA microarray. The four probes in red all mapped to lncRNA HOXD-AS1. **c** The expression levels of HOXD-AS1 in metastatic and non-metastatic HCC tissues. **d** The expression levels of HOXD-AS1 in cancerous and paired adjacent non-cancerous tissues in the metastatic group. **e** The expression levels of HOXD-AS1 in human hepatoma carcinoma cell lines

To identify potential molecular factors associated with intrahepatic metastasis and early recurrence in HCC, we used a lncRNA + mRNA human gene expression microarray to analyze differentially expressed lncRNAs in two groups of HCC tissue samples. Group one contained three samples (with poor prognosis) with intrahepatic metastasis at the time of surgery and was defined as the metastatic liver cancer group. Group two, which was defined as the non-metastatic cancer group, contained three samples (with favorable prognosis) with no evidence of intrahepatic or extrahepatic metastasis at the time of surgery and during the two-year follow-up. As shown in Fig. 1a, hierarchical clustering analysis showed the differential expression of 151 non-coding RNA transcripts (over2-fold, *P* value < 0.01, data not shown) between metastatic and non-metastatic cancer tissues. Among the top-ranked lncRNAs, four probes (Fig. 1b, RNA177677, 177,678, 177,679 and 177,680) mapped to the same lncRNA named HOXD-AS1 (Additional file 1: Figure S1), indicating that HOXD-AS1 was increased by 10-fold in the metastatic group compared to the non-metastatic group. Moreover, by comparing the six cancer tissues in both groups and the respective adjacent non-cancerous tissues in microarray analysis, HOXD-AS1 was also found to be significantly upregulated in cancer tissues (Additional file 1: Figure S1c), suggesting that HOXD-AS1 is involved in liver cancer progression and metastasis.

The expressive pattern of HOXD-AS1 in HCC was further confirmed in a panel of HCC tissue samples (*n* = 44). Consistently, QRT-PCR analysis showed that HOXD-AS1 was highly expressed in metastatic liver cancer tissues compared to non-metastatic cancers (Fig. 1c). Moreover, upregulation of HOXD-AS1 was also observed in most cancer tissues compared to the paired adjacent non-cancerous tissues (Fig. 1d). Additionally, the expression levels of HOXD-AS1 were strongly correlated with the metastatic potentials of four HCC cell lines and the human liver cell line L02 (Fig. 1e), indicating a role for HOXD-AS1 in regulating hepatoma cell metastasis.

### HOXD-AS1 promotes hepatoma cell metastasis and organ colonization

**Fig. 2:**
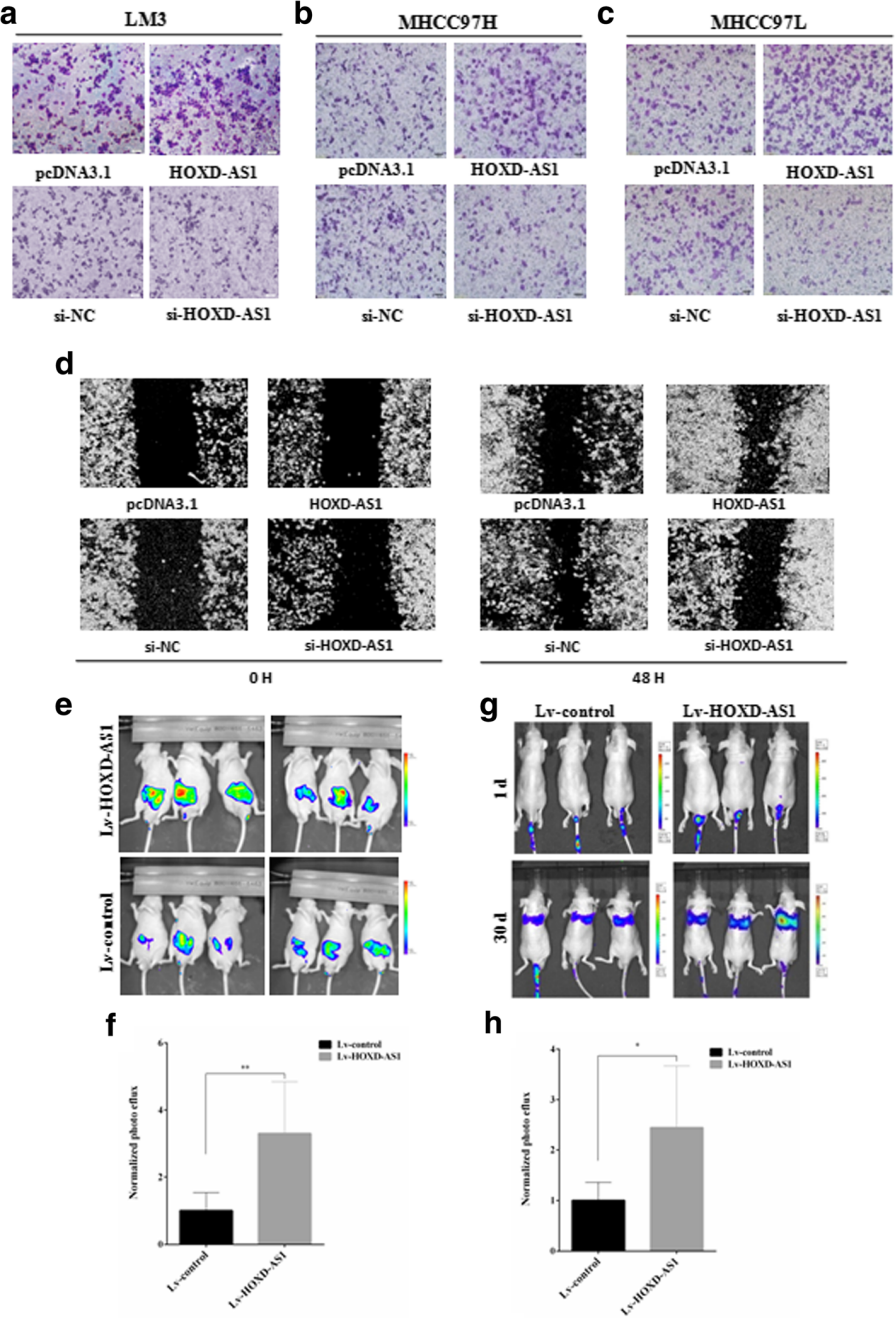
The effects of HOXD-AS1 on metastasis in vitro and in vivo. **a-c** The effects of HOXD-AS1 on HCC cancer cell metastasis were determined by using the Millipore Transwell chambers. Representative images of the bottom surfaces of the chambers with different HCC cells are shown. **d** Scratch wound healing experiments in HOXD-AS1-overexpressing or -silenced HCCLM3 cells. To evaluate the pro-metastatic potential of HOXD-AS1 in vivo, the firefly luciferase-labelled HCCLM3 cells were infected with HOXD-AS1-overexpressing or control lentiviruses for further bioluminescence imaging. **e** The luciferase signal intensities of the mice were examined four weeks after intraspleen injection with 2 × 10^6^ HCCLM3 cells. **f** The luciferase intensity was analyzed in six mice in each group. **g** The luciferase signal intensities of the mice were examined at the indicated time points after tail vein injection with 2 × 10^6^ HCCLM3 cells. **h** The luciferase intensity was analyzed in six mice in each group. The data are shown as the mean ± SD, Student’s t test, *p < 0.05

Nuclear and cytoplasmic RNA were extracted from HCCLM3 cells and the expressive pattern of HOXD-AS1 was detected in both fractions (Additional file 1: Figure S2a). To further explore the biological effect of HOXD-AS1 in HCC metastasis, in vitro transwell assays were performed in HCCLM3, MHCC97H, MHCC97L and L02 cells. Overexpression of HOXD-AS1 significantly augmented the metastatic potential of HCC cancer cells by more than 2-fold (Fig. 2a-c; Additional file 1: Figure S2b), while silencing of HOXD-AS1 strongly inhibited their metastatic potential (Fig. 2a-c; Additional file 1: Figure S2c and d). Next, the pro-metastatic effect of HOXD-AS1 in HCCLM3 cells was also confirmed by wound scratch assays (Fig. 2d). Following the in vitro study, the in vivo behavior of HOXD-AS1 was investigated. Briefly, the firefly luciferase-labelled cells were infected with HOXD-AS1-overexpressing or control lentiviruses and then inoculated into nude mice by intra-spleen injection (Fig. 2e and f) or tail vein injection (Fig. 2g and h). Six weeks after intra-spleen injection of HCCLM3 cells, liver metastasis was more significant when HOXD-AS1 was overexpressed (Fig. 2e and f). Similar results were observed for lung metastasis 30 days after tail vein injection (Fig. 2g and h), suggesting that HOXD-AS1 had a strong pro-metastatic effect in liver cancer.

### HOXD-AS1 accelerates cancer cell growth and inhibits Dox-induced apoptosis

**Fig. 3:**
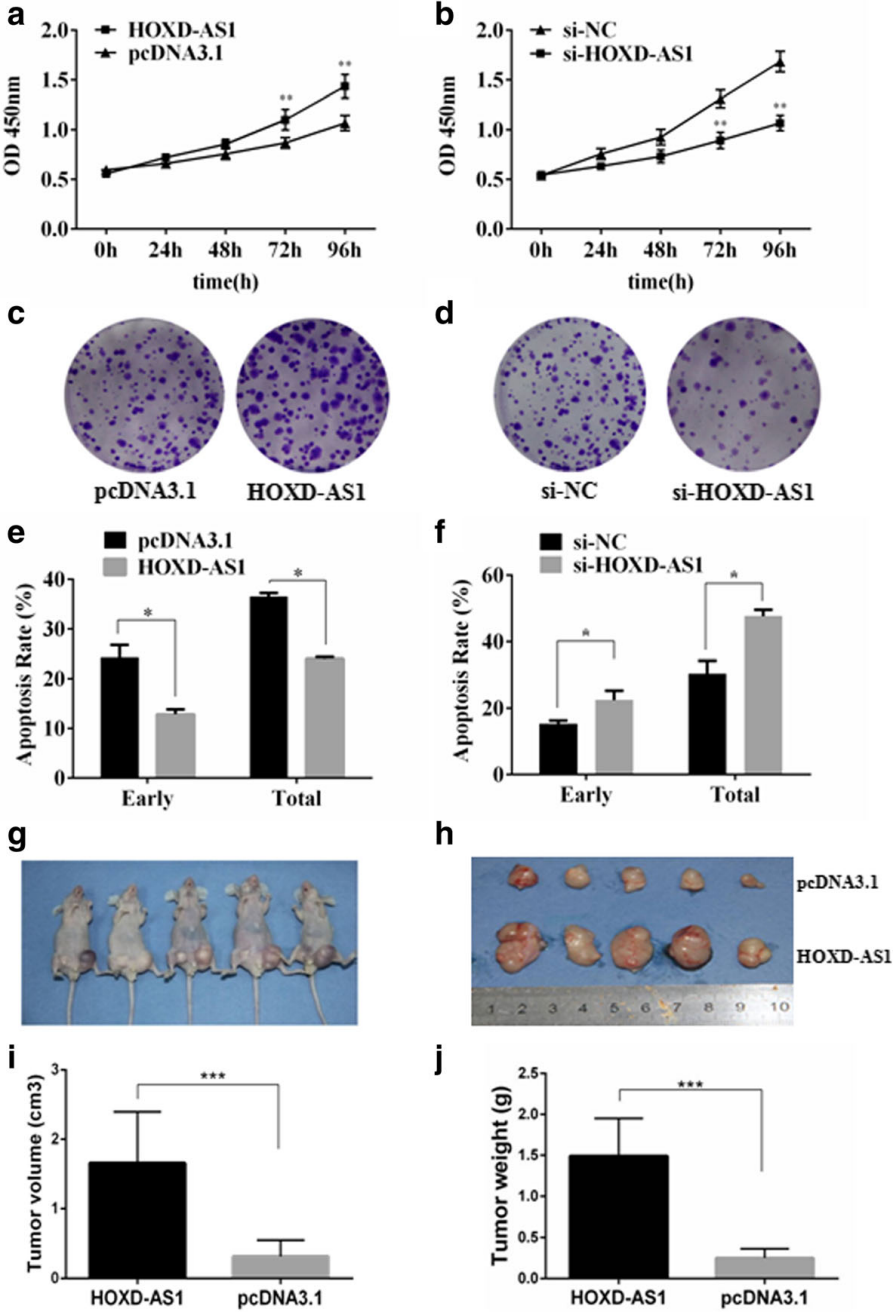
The effect of HOXD-AS1 on cancer cell growth in vitro and in vivo. **a** and **b** MTT assays were performed on HCCLM3 cells with HOXD-AS1 overexpressed or knocked down. **c** and **d** colony formation assays were performed on HCCLM3 cells with HOXD-AS1 overexpressed or knocked down. **e** and **f** HCCLM3 cells were transfected with the HOXD-AS1-pcDNA3.1 overexpression plasmid or siRNAs, and apoptosis was induced by the addition of doxorubicin (Dox,1 μM). Flow cytometry was used to determine the apoptotic rates in the different groups. **g** and **h** Representative xenograft nude mouse model. HCCLM3 cells were transfected with HOXD-AS1-pcDNA3.1 or pcDNA3.1 control vectors and inoculated into the left or right flank of nude mice. **i** Tumor volume and **j** tumor weight were analyzed by the two-sample t test. The data are shown as the mean ± SD, Student’s t test, *p < 0.05, **p < 0.01, ***p < 0.001

As shown in Fig. 1c and d, HOXD-AS1 was overexpressed not only in the metastatic group but also in cancer tissues when compared to the paired non-cancerous tissue, indicating that HOXD-AS1 may participate in both HCC development and progression. Therefore, we investigated the biological function of HOXD-AS1 in cancer cell growth, cell cycle control and apoptosis. Interestingly, MTT assays revealed that HOXD-AS1 overexpression significantly accelerated HCCLM3 cell proliferation, while HOXD-AS1 depletion inhibited the growth of cancer cells (Fig. 3a and b). The pro-proliferative effects of HOXD-AS1 were further determined by colony formation assays, which revealed that the number of colonies was remarkably changed by HOXD-AS1 overexpression or depletion (Fig. 3c and d). However, we did not observe any differences in cell cycle distribution after HOXD-AS1 plasmid or siRNA treatment (Additional file 1: Figure S2e and f), suggesting that HOXD-AS1 may play a regulatory role in cancer cell apoptosis.

Next, the apoptotic rates induced by Dox treatment were evaluated by flow cytometry, which revealed that HOXD-AS1 ectopic overexpression significantly inhibited the early and total apoptotic rates (Fig. 3e; Additional file 1: Figure S2g). HOXD-AS1 depletion by siRNA was able to augment the pro-apoptotic effect of Dox treatment (Fig. 3f; Additional file 1: Figure S2h). Similarly, the mouse xenograft models of HCCLM3 cells showed significant increases in tumor volume and weight by HOXD-AS1 ectopic expression (Fig. 3g-j). In summary, HOXD-AS1 played an essential role in mediating HCC cell growth, apoptosis and metastasis in both the in vitro and in vivo models.

### HOXD-AS1 upregulated ARHGAP11A by competitively binding Mir-19a

To clarify the underlying mechanisms by which HOXD-AS1 promotes metastasis and cell growth in HCC cells, we performed a microarray analysis on HOXD-AS plasmid- or control vector-transfected HCCLM3 cells. The differentially regulated genes were subjected to pathway analysis using GeneMAPP, which revealed several significantly changed pathways (Top 10 pathways, Additional file 1: Figure S3a), including small GTPase-mediated signal transduction, GTPase activator activity, extracellular matrix and regulation of cell proliferation. To identify the potential target of HOXD-AS1, the gene list in Additional file 1: Figure S3a was used for cross comparison between mRNA expression profiles obtained from plasmid-treated HCCLM3 cells and HCC tissues. We found that the expression levels of the Rho GTPase activating protein 11A (ARHGAP11A) and the regulator of G-protein signaling 3 (RGS3) showed great consistency in both in vitro and in vivo mRNA profiling analyses (Additional file 1: Figure S3b). Moreover, HOXD-AS1 can be located within nuclear or cytosolic fractions (Additional file 1: Figure S2a), suggesting that HOXD-AS1 may function through different response regulators via different regulatory mechanisms.

**Fig. 4:**
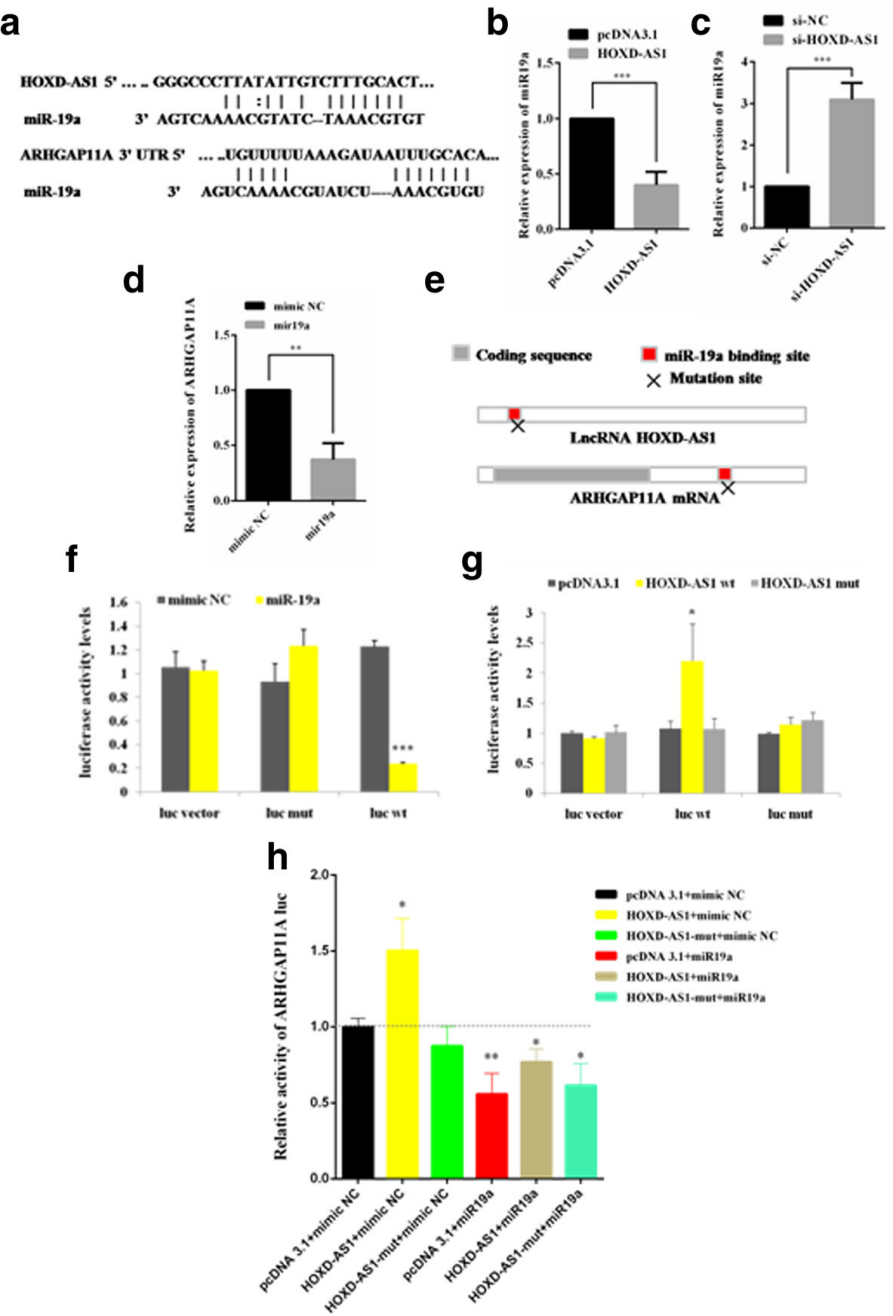
HOXD-AS1 interacts with miR-19a and controls ARHGAP11A expression levels. **a** The prediction for miR-19a binding sites on the HOXD-AS1 transcript and the potential target gene ARHGAP11A. **b** and **c** Relative expression levels of miR19a (compared to U6) after pcDNA3.1-HOXD-AS1 or siRNA treatment measured by QRT-PCR. **d** Relative expression levels of ARHGAP11A (compared to GAPDH) after miR19a or mimic treatment measured by QRT-PCR. **e** Schematic outlining the predicted binding sites and mutation site of miR19a on HOXD-AS1 and the ARHGAP11A transcript. **f** Luciferase activity in HCCLM3 cells cotransfected with miR-19a and empty luciferase reporter or vectors containing the wildtype (luc wt) 3'-UTR region of the ARHGAP11A transcript or mutant (luc wt) transcript. **g** Luciferase activity in HCCLM3 cells cotransfected with wildtype (wt) or mutant (mut) HOXD-AS1 overexpression plasmids and empty luciferase reporter or vectors containing the wildtype (luc wt) 3'-UTR region of the ARHGAP11A transcript or mutant (luc mut) transcript. **h** Luciferase activity in HCCLM3 cells transfected with ARHGAP11A 3'-UTR luciferase reporter vectors, luc wt, luc mut or empty vectors. **f**-**h** The data are presented as the relative ratio of firefly luciferase activity to renilla luciferase activity. The data are shown as the mean ± SD. Student’s t test, *p < 0.05, **p < 0.01, ***p < 0.001

The lncRNA-miRNA and mRNA-miRNA interactions are commonly involved in various biological processes. Using an online database (http://starbase.sysu.edu.cn/), we found that HOXD-AS1 has one predicted miR19a target binding site (Fig. 4a) [23, 24]. Moreover, by comparing the miR19a target gene panel and the mRNA profiling data from the lncRNA+ mRNA human gene expression microarray analysis, we found that ARHGAP11A was significantly upregulated in metastatic HCC tissue samples and was predicted to be a target gene of miR19a (Fig. 4a). We hypothesized that HOXD-AS1 functions as a competing endogenous RNA (ceRNA) by “sponging” miR19a during HCC metastasis/recurrence. To test this hypothesis, we first demonstrated that miR19a was repressed or induced by HOXD-AS1 overexpression or knockdown (Fig. 4b and c), respectively, and that the expression levels of ARHGAP11A were downregulated by miR19a mimic treatment as well (Fig. 4d). Second, the 3′-UTR region of ARHGAP11A was inserted into a luciferase reporter system containing wild-type (wt) or mutated miR19a binding sites (Fig. 4e). MiR19a was able to repress the luciferase activity of the wt reporter vector but had no effect on the mutant vector (Fig. 4f). Third, we also constructed a pcDNA3.1-HOXD-AS1-mut vector, in which the “sponging” sites for miR19a were mutated. Overexpression of HOXD-AS1 (wt) increased the luciferase activity of the ARHGAP11A 3′-UTR (wt) containing vector; however, the pcDNA3.1-HOXD-AS1-mut treatment group showed no changes (Fig. 4g). Additionally, ectopic expression of miR-19a abrogated the upregulating effects of HOXD-AS1 (wt) on the ARHGAP11A 3′-UTR (wt) containing luciferase activity (Fig. 4h). Therefore, HOXD-AS1 could act as a ceRNA via its miR19a binding site and could upregulate the expression of ARHGAP11A in HCC cells.

### ARHGAP11A downregulation repressed hepatoma cell metastasis and was correlated with better outcomes in HCC patients

**Fig. 5:**
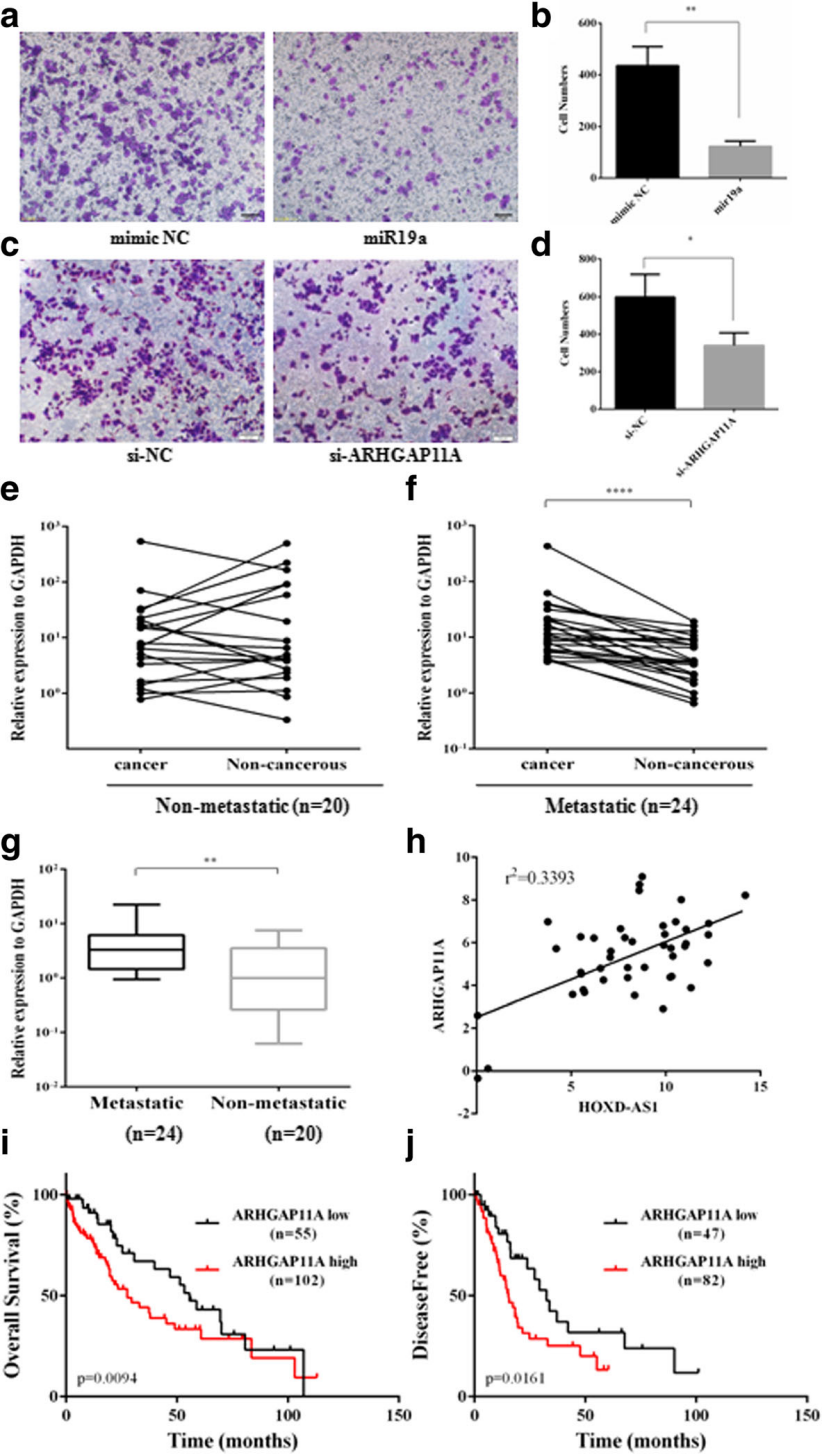
ARHGAP11A expression contributes to the metastatic phenotype of HCC. **a-d** Transwell assays on miR19a-treated or ARHGAP11A-silenced HCCLM3 cells. **e** and **f** The expression levels of ARHGAP11A in cancerous and paired adjacent non-cancerous tissues in the non-metastatic and metastatic groups. **g** The expression levels of ARHGAP11A in non-metastatic and metastatic HCC tissues. **h** The correlation between HOXD-AS1 transcript levels and ARHGAP11A mRNA levels was measured in 44 HCC tissues. The dCt values (normalized to GAPDH) were subjected to Pearson correlation analysis. **i** and **j** The prognostic significance of ARHGAP11A in HCC patients. The Caner Genome Atlas project (TCGA) dataset was analyzed to reveal the effect of ARHGAP11A mRNA levels on the overall survival (OS) or disease-free survival (DFS) of patients with HCC using cBioPortal (http://cbioportal.org)

Because HOXD-AS1 was able to upregulate ARHGAP11A expression in vitro, we next asked whether the miR19a/ARHGAP11A axis contributes to HOXD-AS1-mediated cancer cell metastasis. Notably, ectopic expression of miR19a strongly decreased cell migration by over 2-fold (Fig. 5a and b, *p*<0.01). This anti-metastatic effect of miR19a was in accordance with a previous report by Han and his colleagues showing that miR19a was significantly downregulated in a group of HCC samples from patients who developed recurrent liver cancer compared to those with non-recurrence. The expression levels of miR19a were correlated with patient survival with a hazard ratio of 0.724 (*p* = 0.02) [25]. On the other hand, siRNA knockdown of ARHGAP11A markedly decreased cell migration compared to the control (Fig. 5d and d), indicating that ARHGAP11A had a pro-metastatic effect.

We next examined whether ARHGAP11A was aberrantly expressed in HCC tissue samples. However, we found no obvious differences in its expression between paired non-metastatic cancer and adjacent non-cancerous tissues (Fig. 5e). On the other hand, ARHGAP11A was significantly overexpressed in cancer tissue compared to non-cancerous tissue in the metastatic group (Fig. 5f). Intriguingly, a significant upregulation of ARHGAP11A was observed in metastatic cancers compared to the non-metastatic cancer tissues (Fig. 5g, *p*<0.01). Moreover, to examine whether HOXD-AS1 was co-expressed with ARHGAP11A in HCC samples, the expression levels of both genes were measured in the panel of 44 HCC tissue samples. As shown in Fig. 5h, there was a significant positive correlation between the expression levels of HOXD-AS1 and ARHGAP11A (r^2^ = 0.3393). In addition, clinicopathological analysis revealed that the overexpression of both genes was closely correlated with Grades III and IV (HOXD-AS1, *p* = 0.0381; ARHGAP11A, *p* = 0.0006) and with PVTT tumor invasion (HOXD-AS1, *p* = 0.0419; ARHGAP11A, *p* = 0.0079) (Additional file 1: Table S3).

To explore the clinical significance of ARHGAP11A, we also utilized The Cancer Genome Atlas (TCGA) dataset and analyzed the association between mRNA expression Z-scores and patient survival (Fig. 5i and j). The Kaplan–Meier survival analyses demonstrated that HCC cancer patients with lower ARHGAP11A mRNA expression exhibited better prognosis compared with those patients with higher ARHGAP11A expression (Gehan-Breslow-Wilcoxon test, *p* = 0.0094, log-rank test, *p* = 0.059). The median overall survival of the high and low expression groups was 27.5 months and 55.6 months, respectively (Fig. 5i). We also observed a significant difference in disease-free survival, measured in months, between the two groups (Fig. 5j). The high expression group showed poor prognosis compared to the low expression group (Gehan-Breslow-Wilcoxon test, *p* = 0.0161, log-rank test, *p* = 0.0182), with median disease-free survival of 15.4 months and 32.5 months, respectively.

### Downregulation of RGS3 expression by HOXD-AS1 contributes to the inhibition of Dox-induced apoptosis

**Fig. 6:**
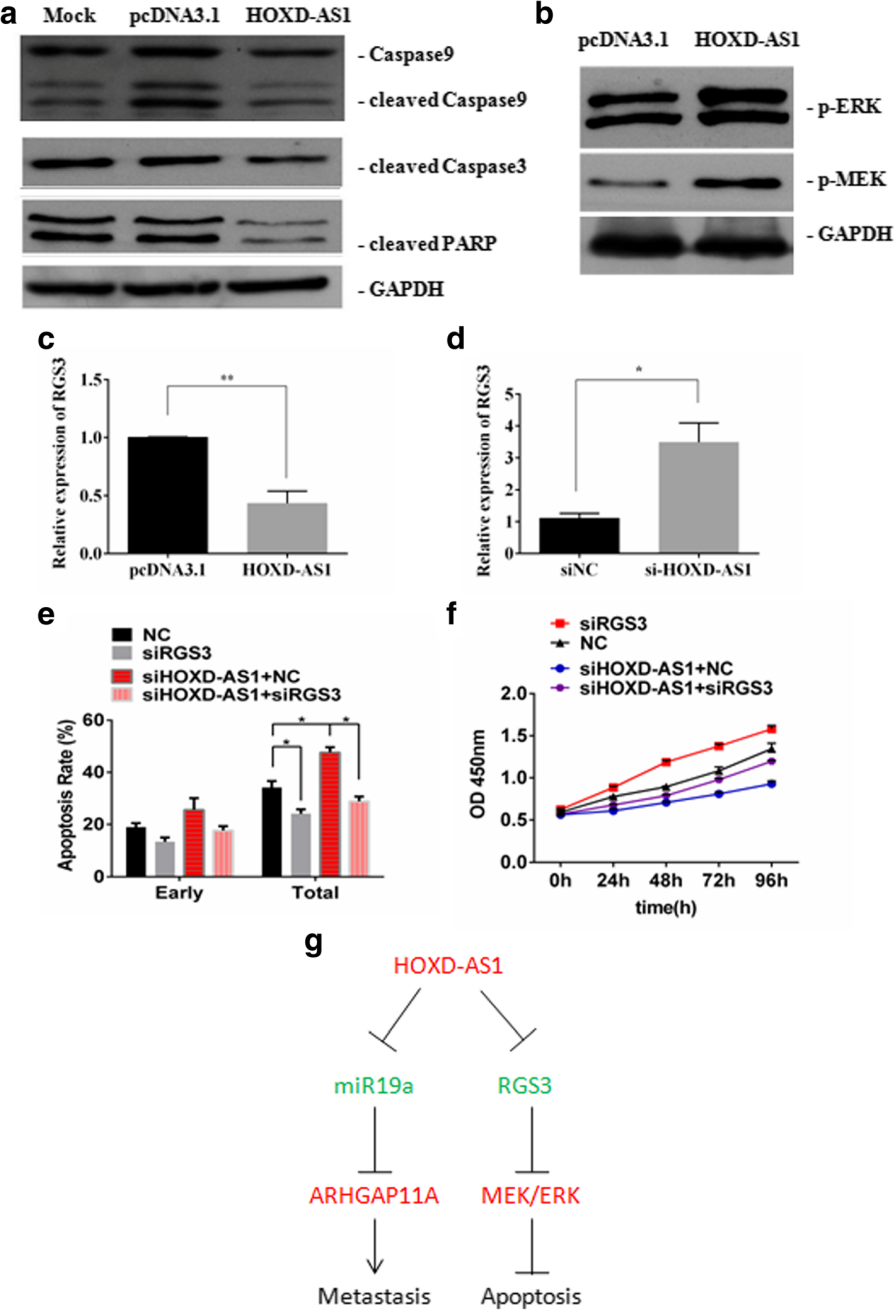
HOXD-AS1 downregulated RGS3 expression and inhibited Dox-induced apoptosis. **a** The protein expression levels of cleaved caspase9, cleaved caspase3 and cleaved PARP were determined by westernblot analysis after Dox treatment. **b** Quantification by westernblot of phosphorylated ERK and MEK in HCCLM3 cells when HOXD-AS1 was overexpressed compared to pcDNA3.1 empty vector. **c** and **d** Quantification of RGS3 mRNA expression by QRT-PCR when HOXD-AS1 was overexpressed or silenced by siRNA. **e** HCCL3 cells were treated with siRNA targeting HOXD-AS1 or RGS3 followed by examination of Dox-induced apoptosis by flow cytometry. **f** MTT assays were performed after siRNA (siNC, si-HOXD-AS1 or si-RGS3) treatment. **g** Schematic model of HOXD-AS1 functions during liver cancer progression

Next, we examined the activation and cleavage of caspases and poly (ADP-ribose) polymerase (PARP) to identify molecular events that could account for the inhibited Dox-induced apoptosis by HOXD-AS1 overexpression (Fig. 3e and f). Dox, as a broad-spectrum antitumor anthracycline antibiotic commonly used for the treatment of HCC, either by systemic delivery or by means of transcatheter arterial chemoembolization (TACE), [26] has been shown to induce caspase activation and apoptosis in cancer cells. In fact, we observed that the overexpression of HOXD-AS1 significantly abrogated the cleavage of caspase 9, caspase 3 and PARP in HCCLM3 cells treated by Dox (Fig. 6a), reaffirming the anti-apoptotic role of HOXD-AS1 in HCC cells. Since miR19a inhibitor treatment did not show anti-apoptotic effect (Additional file 1: Figure S4a and b), we presumed that the anti-apoptotic effect of HOXD-AS1 was not induced by miR19a inhibition when HOXD-AS1 was overexpressed. The microarray data and GeneMAPP analysis revealed that HOXD-AS1 upregulation led to significant changes in genes that activate or inactivate the activity of GTPases (Additional file 1: Figure S3), which are a large group of key regulators that participate in signal transduction through their ability to cycle between an active GTP-bound state and an inactive GDP-bound state. Interestingly, we noted a significant activation of the MEK/ERK signaling cascade after HOXD-AS1 overexpression (Fig. 6b). More importantly, we identified that RGS3, a potential negative regulator of MEK/ERK signaling, [27] was downregulated or upregulated by HOXD-AS1 overexpression or siRNA treatment, respectively (Fig. 6c and d). Further apoptotic assays by flow cytometry showed that the depletion of RGS3 by siRNA strongly inhibited Dox-induced apoptosis and largely abrogated the increased apoptotic rate caused by the HOXD-AS1 knockdown (Fig. 6e). Additionally, MTT assays revealed that the RGS3 knockdown also led to a significant increase in cell proliferation (Fig. 6f). Based on the above evidence, we propose that inhibition of RGS3 expression by HOXD-AS1 contributes to the suppression of Dox-induced apoptosis.

## Discussion

Metastasis and the development of de novo HCC are two main causes for the poor prognosis of cancer patients [3]. In the present study, we directly screened metastatic HCC tissue samples and non-metastatic HCC samples and identified a significantly upregulated lncRNA, HOXD-AS1, in the metastatic group (Fig. 1; Additional file 2: Table S5). HOXD-AS1 was also overexpressed in most cancerous tissue compared to the paired adjacent non-cancerous tissue (Fig. 1 and Additional file 1: Figure S1). Moreover, clinicopathological analysis revealed that overexpression of HOXD-AS1 was closely correlated with higher tumor stage and PVTT tumor invasion (Additional file 1: Table S3), indicating that HOXD-AS1 is a potential oncogene in HCC progression and metastasis. Furthermore, the pro-metastatic effect of HOXD-AS1 was demonstrated by in vitro transwell and wound scratch assays and by in vivo xenograft mouse model experiments (Figs. 1 and 2).

The underlying mechanisms by which HOXD-AS1 promoted cancer cell metastasis were then investigated. Recently, several lncRNAs were reported to act as ceRNAs by competitively binding microRNAs. For instance, linc-MD1 upregulates the expression of two transcription factors (TFs) that control muscle-specific gene expression by competitively binding to miR-133 [28]. LncRNA-activated by TGF-β (lncRNA-ATB) “sponges” miR-200 family to regulate the expression of ZEB1 and ZEB2 and then induces EMT [19]. Our data indicated that HOXD-AS1 could also function as a ceRNA by “sponging” miR19a, and that it could increase the expression levels of the miR19a target gene, ARHGAP11A (Fig. 4). Indeed, aberrant expression of mir19a, a possible oncogene belonging to the miR-17-92 cluster, was previously reported in multiple cancers, such as lung, colon and gastric cancers [29–31]. However, a recent clinical study of 165 HCC patients revealed a differential role for miR-19a in cancer metastasis/recurrence in which miR19a was significantly downregulated in recurrent HCCs, and the miR19a downregulation was correlated with patient survival with a hazard ratio of 0.724, suggesting that miR19a is an anti-oncogene in HCC metastasis/recurrence [25]. Consistently, our data revealed that downregulation of miR19a expression correlated well with higher tumor stage (*p* = 0.0023) and PVTT tumor invasion (*p* = 0.0448) (Additional file 1: Table S4). Therefore, our investigation provides a possible explanation for miR19a downregulation contributing to liver cancer progression, specifically the upregulation of the miR19a target gene, ARHGAP11A (Fig. 4). We only noticed the decrease of miR19a in a number of cancer tissues compared to the adjacent non-cancerous tissues in the metastatic group (Additional file 1: Figure S4 c-e); however, the differences in gene expression were not significant. Future studies with a larger sample size will be helpful in clarifying the biological significance of miR19a in HCC metastasis and progression.

The Ras homology (Rho) subfamily of small GTPases represents a family of small GTP-binding proteins involved in cell migration, cytoskeleton organization and proliferation. These proteins are emerging as a new class of biomarkers for cancer prognosis [32–34]. ARHGAP11A belongs to the Rho GTPase activation protein (RhoGAP) subfamily, members of which promote the hydrolysis of GTP and inactivate Rho GTPases. The role of ARHGAP11A in cancer, however, is controversial. Xu and his colleagues demonstrated that ARHGAP11A accumulated in the nuclei of colorectal cancer cells, interacted with p53 and then induced apoptosis [35]. In another study evaluating colorectal cancer development, Kagawa et al. showed that ARHGAP11A was significantly upregulated in colon cancers and that its expression levels correlated with clinical invasion status, which may result from suppression of RhoA and increased Rac1 activity [36]. Our investigation provided evidence supporting the oncogenic and pro-metastatic effects of ARHGAP11A (Fig. 5), consistent with Kagawa’s observation.

We also examined the biological relevance of HOXD-AS1 in cancer cell growth in vitro and in vivo. This analysis revealed that HOXD-AS1 overexpression remarkably decreased Dox-induced apoptosis in HCC (Fig. 3e and f). Intriguingly, HOXD-AS1 also activates the best known G protein coupled receptor signal transduction MEK/ERK signal cascade (Fig. 6b), accompanied by decreased expression of RGS3 (Fig. 6c), which is a potential negative regulator of MEK/ERK signaling [27]. RGS proteins serve as GTPase-activating proteins for heterotrimeric G proteins and, therefore, inactivate G protein-coupled receptor signaling pathways [27]. Previous studies reported that several RGS proteins, including RGS2, RGS4, and RGS5, were involved in cancer development [37–39]. As a member of the RGS family, RGS3 controls the signaling mediated by the Gα_q_ and Gα_i_ proteins by binding to the corresponding Gα subunits of heterotrimeric G proteins, which may cause changes in the activities of ERK, JNK and p38MAPK [40]. Moreover, RGS3 inhibits the activation of MAPK and Akt via Gβγ subunits [41]. Additionally, RGS3 is known to protect against cardiac hypertrophy by blocking the MEK/ERK signaling pathway [27]. Therefore, we presumed that HOXD-AS1 may activate MEK/ERK signaling by repressing the expression levels of RGS3, which contributes to the inhibited apoptosis and accelerated proliferation during HCC progression. It has been reviewed that lncRNAs work through multiple mechanisms including ceRNAs, chromatin remodeling and natural antisense transcripts; and some lncRNAs even possess multiple mechanism characteristics, [42, 43]. How HOXD-AS1 represses RGS3 expression in HCC cells still remain elusive. Since HOXD-AS1 can be located within nuclear or cytosolic fractions, we presumed that HOXD-AS1 may play a role in chromatin remodeling.

## Conclusions

In summary, our investigation has presented evidence for the essential role of the lncRNA HOXD-AS1 in liver cancer progression and metastasis (Fig. 6g). More importantly, the discovery of the HOXD-AS1/miR19a/ARHGAP11A signaling axis has provided new knowledge for understanding the molecular basis of liver cancer and for the development of new diagnostic and therapeutic strategies.

## Additional files

Additional file 1: Table S1. siRNAs and QRT-PCR primers. **Table S2.** QRT-PCR primers. **Figure S1.** Overexpression of HOXD-AS1 in HCC tissue samples. **Figure S2.** The biological roles of HOXD-AS1 in HCC. **Figure S3.** Potential downstream effectors of HOXD-AS1. **Table S3.** Correlation between gene expression and clinicalpathological characteristics. **Figure S4.** Biological roles of miR-19a in liver cancer. **Table S4. **Correlation between miR-19a expression and clinicalpathological characteristics. (PDF 732 kb)

Additional file 2: Table S5. Data analysis of lncRNA microarray. (XLSX 191 kb)

## Abbreviations

ARHGAP11A: Rho GTPase activating protein 11A
Dox: Doxorubicin
HCC: Human hepatocellular carcinoma
HOTAIR: Hox transcript antisense intergenic RNA
HOTTIP: HOXA transcript at the distal tip
HOXD-AS1: HOXD cluster antisense RNA 1
lncRNA-ATB: lncRNA-activated by TGF-β
lncRNA-Dreh: lncRNA downregulated expression by HBx
lncRNA-LET: lncRNA low expression in tumor
lncRNAs: long noncoding RNAs
MALAT1: Metastasis-associated lung adenocarcinoma transcript-1
miR-19a: microRNA-19a
PVTT: Portal vein tumor thrombi
TCGA: The Cancer Genome Atlas
TGF-β: Transforming growth factor-β
VEGF: Vascular endothelial growth factor
