# Supplementary material for: The noncoding RNA HOXD-AS1 is a critical regulator of the metastasis and apoptosis phenotype in human hepatocellular carcinoma
Source: Mol Cancer. 2017 Jul 19;16:125. doi: 10.1186/s12943-017-0676-x (PMC5518122; doi:10.1186/s12943-017-0676-x)
Supplement: Supplementary file 1 — siRNAs and QRT-PCR primers. Table S2. QRT-PCR primers. Figure S1. Overexpression of HOXD-AS1 in HCC tissue samples. Figure S2. The biological roles of HOXD-AS1 in HCC. Figure S3. Potential downstream effectors of HOXD-AS1. Table S3. Correlation between gene expression and clinicalpathological characteristics. Figure S4. Biological roles of miR-19a in liver cancer. Table S4. Correlation between miR-19a expression and clinicalpathological characteristics. (PDF 732 kb) [file 12943_2017_676_MOESM1_ESM.pdf]

Table S1. siRNAs and QRT-PCR primers

Name		Sequence
NC	Sense	5'-UUCUCCGAACGUGUCACGUTT-3'
	Anti-sense	5'-ACGUGACACGUUCGGAGAATT-3'
HOXD-AS1	Sense	5'-GACCUUCCUUAGACCUAUAAC-3'
	Anti-sense	5'-UAUAGGUCUAAGGAAGGUCCU-3'
HOXD-AS1-1	Sense	5'-AGAGGGAAGUUUCAGCUAAUU-3'
	Anti-sense	5'-UUAGCUGAAACUUCCCUCUGA-3'
GAPDH	Sense	5'-GUAUGACAACAGCCUCAAGTT-3'
	Anti-sense	5'-CUUGAGGCUGUUGUCAUACTT-3'
RGS3	Sense	5'- GACAGUGCAGACCAUGAAGTT-3'
	Anti-sense	5'- CUUCAUGGUCUGCACUGUCTT-3'
ARHGAP11A	Sense	5'-TCTTAGATCCAGTGAGAATTT-3'
	Anti-sense	5'-ATTCTCACTGGATCTAAGATT-3'

Table S2. QRT-PCR primers

Name	Sequence
Human-β-actin-RT-F	TGTGTTGGCGTACAGGTCTTTG
Human-β-actin-RT-R	GGGAAATCGTGCGTGACATTAAG
Human-18sRNA-RT-F	CGTTCTTAGTTGGTGGAGCG
Human-18sRNA-RT-R	CGCTGAGCCAGTCAGTGTAG
HOXD-AS1-RT-F	TCTGAAAGAAGGACCAAAGTAA
HOXD-AS1-RT-R	ATTCAAGGGACAGTCACAGG
Human-RGS3-F	CGCTTTCTCCGTTCTGACCTCT
Human-RGS3-R	GCTTCCGTATGTCTATCCGTCTGTC
Human- ARHGAP11A-F	AAGCGGGTCTGTGTAAGTGG
Human- ARHGAP11A-R	CCTCTGATCCCACATTCCGG
Human- GAPDH-F	GAGTCAACGGATTTGGTCGT
Human- GAPDH-R	GACAAGCTTCCCGTTCTCAG

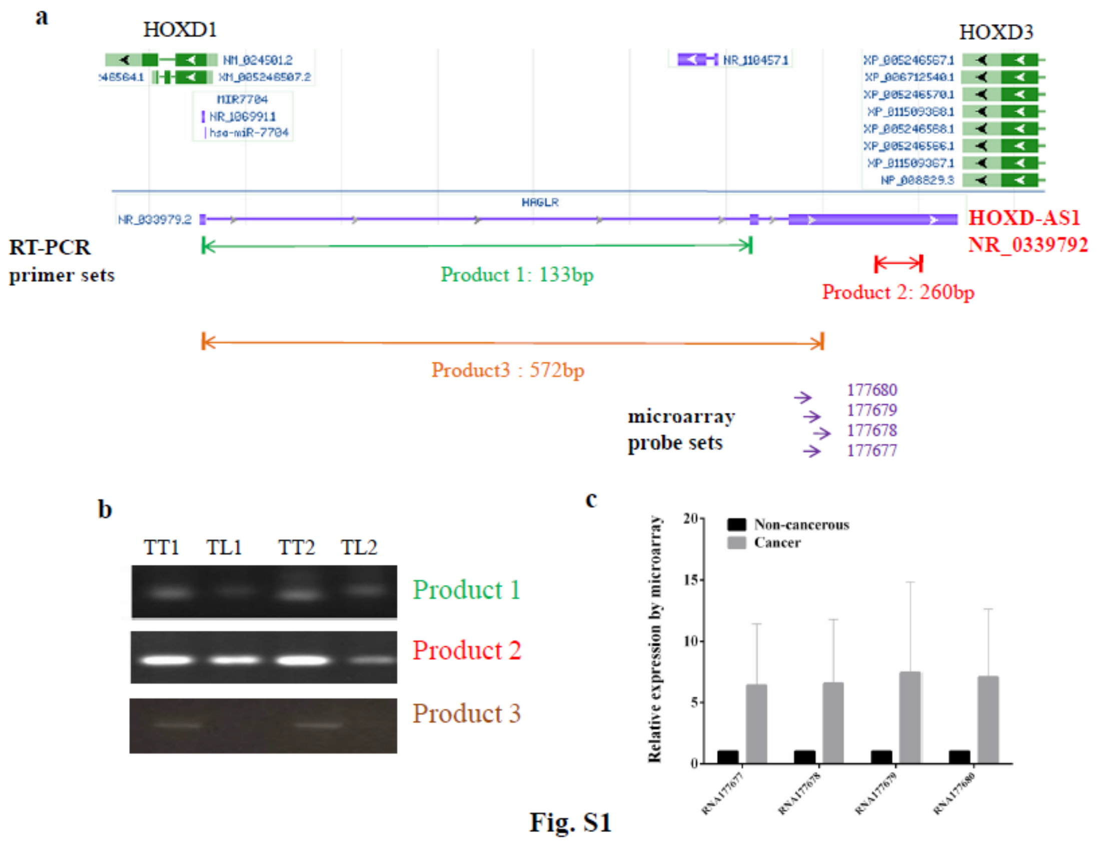

Fig. S1

Fig. S1. Overexpression of HOXD-AS1 in HCC tissue samples. **a** Positions of different primer pairs targeting different annotated exons mapped in NCBI relative to HOXD-AS1 RefSeq NR_033979.2. For lncRNA + mRNA microarray analysis, probe sets were shown in purple. **b** RT-PCR detection of PCR product using primer 1-3 in metastatic tumor (TT) or paired non-tumor (TL) tissue. **c** The signal intensities of the probe set of HOXD-AS1 in six paired cancer and non-cancerous tissue samples detected by lncRNA + mRNA microarray analysis.

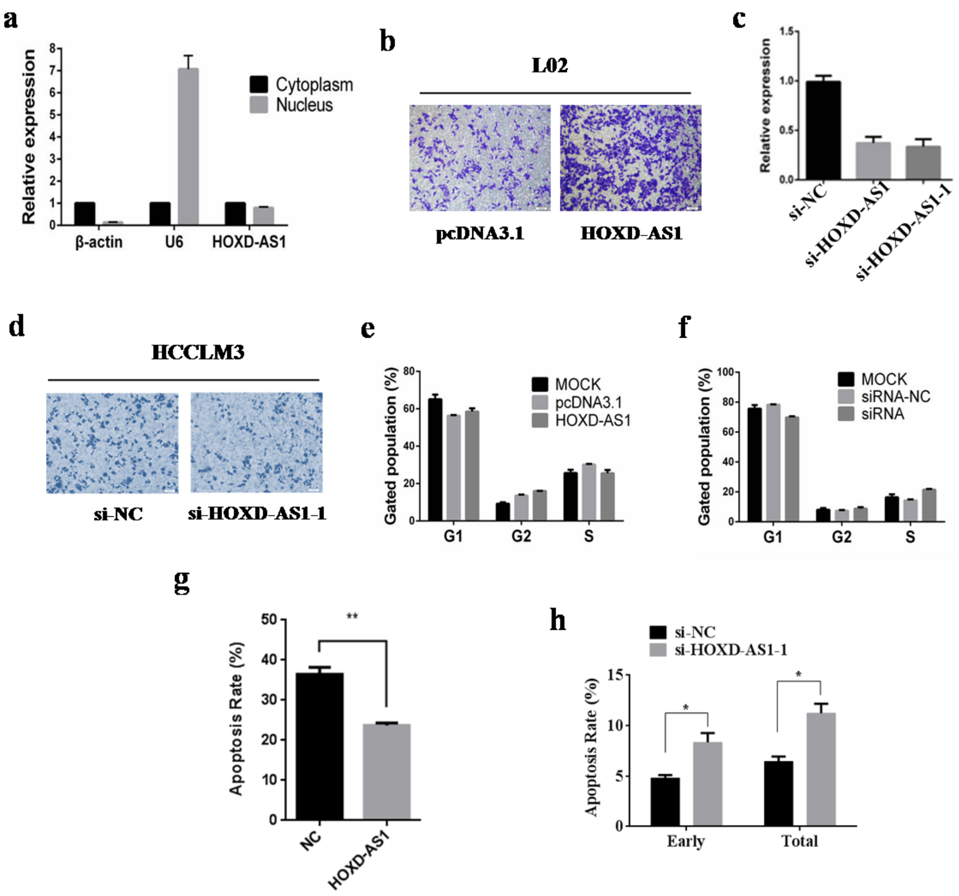

Fig. S2

Fig. S2. The biological role of HOXD-AS1 in HCC. **a** Nuclear and cytoplasmic RNA were extracted from HCCLM3 cells and the expression of HOXD-AS1 was analyzed in both fractions. **b** The effects of HOXD-AS1 overexpression on L02 cell metastasis were determined by using the Millipore Transwell chambers. **c** HOXD-AS1 was knocked down by two independent siRNAs. **d** The metastatic potential of HCCLM3 cells was inhibited via another siRNA sequence, si-HOXD-AS1-1. **e** and **f** The effect of HOXD-AS1 overexpression or knockdown in cell cycle distribution in HCCLM3 cells were analyzed by flow cytometry 72 hours after transfection. **g** Apoptotic rates in L02 cells were determined by flow cytometry after HOXD-AS1 overexpression. **h** Apoptotic rates in HCCLM3 cells were determined by flow cytometry after si-HOXD-AS1-1 treatment.

a

Pathway	Count	p-Value	Gene
Small GTPase mediated signal transduction	8	369E-07	IGF1; ARHGAP5; ARL5A; NOTCH2; RAB28; RAP2A; RAPGEF2; RAPGEF6
Enzyme activator activity	7	298E-06	RASA4; **RGS3**; UBA7; **ARHGAP11A**; ARHGAP5; RABGAP1; RAPGEF2
Enzyme binding	6	166E-06	AKAP12; DOCK11; DOCK2; DOCK8; AKAP2; RAPGEF6
Small GTPase regulator activity	6	199E-06	DOCK11; DOCK2; DOCK8; ARHGAP5; RAPGEF2; RAPGEF6
GTPase activator activity	6	455E-06	RASA4; **RGS3**; **ARHGAP11A**; ARHGAP5; RABGAP1; RAPGEF2
Cellular morphogenesis	6	571E-06	CD3G; OSM; AKAP2; CAP2; NOTCH2; SOCS5
Kinase activity	6	614E-06	PLAU; ALPK2; AKAP2; PKN2; WDR65; IPMK
Extracellular matrix	6	114E-05	ADAMTS4; COL8A1; LTBP2; MFAP4; SMOC2; DMP1
Regulation of cell proliferation	6	724E-05	AZGP1; ADAM33; IGF1; OSM; PTHLH; NOTCH2
Extracellular space	6	128E-04	PLAU; ANGPTL2; CHIT1; LTBP2; OSM; PTHL

b

Gene Symbol	LM3 (mRNA microarray) HOXD-AS1 vs pcDNA31	HCC tissue (lncRNA + mRNA microarray)	
		Metastatic/ non-metastatic	Cancer/ non-cancerous
ARHGAP11A	231 (up)	259 (up)	167 (up)
RGS3	037 (down)	056 (down)	033 (down)

Fig. S3

Fig. S3. Potential downstream effectors of HOXD-AS1. **a** HOXD-AS1 overexpression or empty plasmids were transfected into HCCLM3 cells and then total RNA was extracted and analyzed by mRNA microarray analysis. Differentially regulated genes were subjected to GeneMAPP analysis and signal pathways containing the top 10 largest number of genes were listed. RGS3 and ARHGAP11A (red) were studied in the present investigation as the downstream effectors of HOXD-AS1. **b** ARHGAP11A and RGS3 expression in HCC tissue and cell samples. In HCCLM3 cells, HOXD-AS1 or pcDNA3.1 plasmids were transfected and cells were collected after 72 h for mRNA microarray analysis. For HCC tissue samples, the expression levels of ARHGAP11A and RGS3 were compared across different sets.

Table S3 Correlation between gene expression and clinicalpathological characteristics

Variables		HOXD-AS1 expression				ARHGAP11A expression			
		Overexpression n (%)	Normal expression n (%)	Chi-square	P value	Overexpression n (%)	Normal expression n (%)	Chi-square	P value
Age									
	<55	6 (42.9%)	2 (28.6%)		0.6657	4 (30.8%)	4 (50%)		0.6458
	≥55	8 (57.1%)	5 (71.4%)			9 (69.2%)	4 (50%)		
Gender									
	Male	15 (88.2%)	5 (83.3%)		n.s.	12 (92.3%)	8 (80%)		0.5596
	Female	2 (11.8%)	1 (16.7%)			1 (7.7%)	2 (20%)		
HBV									
	Negative	3 (17.6%)	0 (0%)		0.5296	1 (6.3%)	2 (25%)		0.2490
	Positive	14 (82.4%)	7 (100%)			15 (93.7%)	6 (75%)		
Disease Stage									
	Ⅰ and Ⅱ	2 (11.8%)	7 (100%)		<0.001***	0 (0%)	9 (100%)		<0.001***
	Ⅲ and Ⅳ	15 (88.2%)	0 (0%)			15 (100%)	0 (0%)		
Serum AFP concentration									
	<20μg/L	6 (35.3%)	2 (28.6%)		0.6241	4 (25%)	4 (50%)		0.3625
	≥20μg/L	11 (64.7%)	5 (71.4%)			12 (75%)	4 (50%)		
Portal vein thrombosis									
	No	9 (33.3%)	11 (64.7%)	4.141	0.0419*	8(29.6%)	12 (70.6%)	7.059	0.0079**
	Yes	18 (66.7%)	6 (35.3%)			19 (70.4%)	5 (29.4%)		

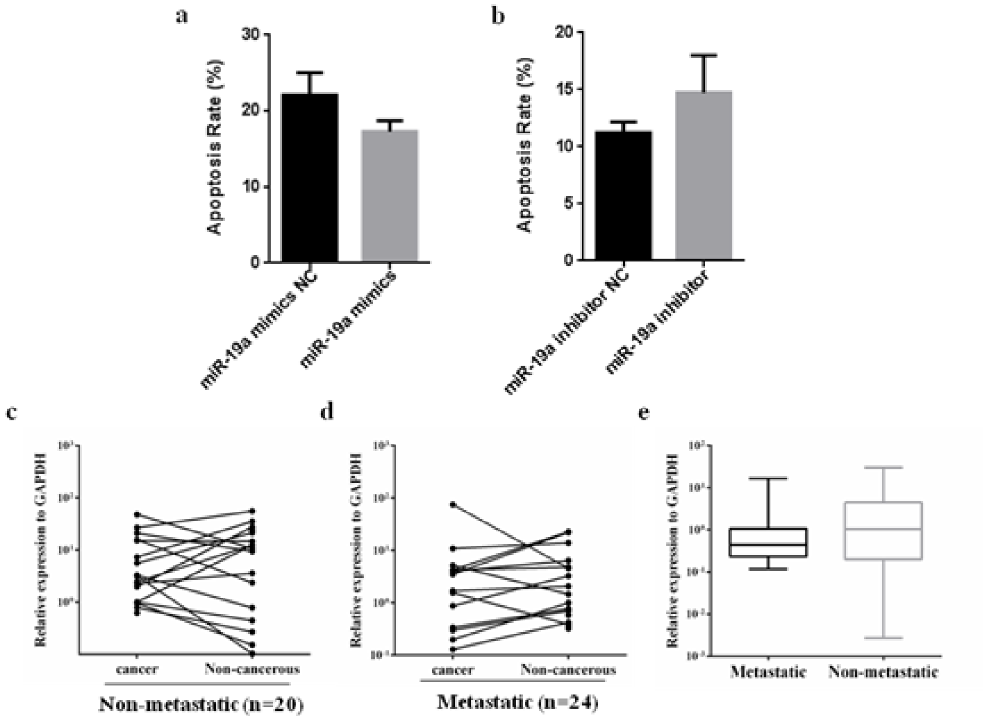

Fig. S4

Fig. S4. Biological roles of miR-19a in liver cancer. **a** and **b** HCCLM3 cells were treated with miR-19a mimics or inhibitor, apoptosis was induced by the addition of doxorubicin (Dox,1 μM). Flow cytometry was used to determine the apoptotic rates in the different groups. **c-e** Expression levels of miR-19a in Non-metastatic **c**,metastatic **d** or both groups **e**.

Table S4 Correlation between miR-19a expression and clinicalpathological characteristics

Variables		Down expression n (%)	Normal expression n (%)	Chi-square	P value
Age					
	<55	5 (45.5%)	3 (33.3%)		0.6699
	≥55	6 (54.5%)	6 (66.7%)		
Gender					
	Male	12 (92.3%)	7 (77.8%)		0.5442
	Female	1 (7.7%)	2 (22.2%)		
HBV					
	Negative	1 (7.7%)	1 (10%)		n.s.
	Positive	12 (92.3%)	9 (90%)		
Disease Stage					
	Ⅰ and Ⅱ	1 (7.1%)	6 (50%)		0.0023**
	Ⅲ and Ⅳ	13 (92.9%)	2 (50%)		
Serum AFP concentration					
	<20μg/L	4 (30.8%)	3 (33.3%)		n.s.
	≥20μg/L	9 (69.2%)	6 (66.7%)		
Portal vein thrombosis					
	No	8 (66.7%)	11 (73.3%)		0.0448*
	Yes	14 (33.3%)	4 (26.7%)		

P values of Fisher's exact test.
